# Corticotrophin-Releasing Factor Modulates Cerebellar Purkinje Cells Simple Spike Activity *in Vivo* in Mice

**DOI:** 10.3389/fncel.2018.00184

**Published:** 2018-07-05

**Authors:** Hong-Wei Wang, Jing-Tong Zhao, Bing-Xue Li, Shan-Shan Su, Yan-Hua Bing, Chun-Ping Chu, Wei-Ming Wang, Yu-Zi Li, De-Lai Qiu

**Affiliations:** ^1^Department of Cardiology, Affiliated Zhongshan Hospital of Dalian University, Dalian, China; ^2^Key Laboratory of Cellular Function and Pharmacology of Jilin Province, Yanbian University, Yanji, China; ^3^Department of Cardiology, Affiliated Hospital of Yanbian University, Yanji, China; ^4^Department of Physiology and Pathophysiology, College of Medicine, Yanbian University, Yanji, China; ^5^Department of Osteology, Affiliated Zhongshan Hospital of Dalian University, Dalian, China; ^6^Key Laboratory of Natural Resource of the Changbai Mountain and Functional Molecular of the Ministry of Education, Yanbian University, Yanji, China

**Keywords:** cerebellar Purkinje cell, *in vivo* whole-cell patch-clamp recording, complex spike (CS), corticotropin-releasing factor (CRF), miniature postsynaptic currents, simple-spike (SS), protein kinase A (PKA)

## Abstract

Corticotropin-releasing factor (CRF) is a major neuromodulator that modulates cerebellar neuronal activity via CRF receptors during stress responses. In the cerebellar cortex, CRF dose-dependently increases the simple spike (SS) firing rate of Purkinje cells (PCs), while the synaptic mechanisms of this are still unclear. We here investigated the effect of CRF on the spontaneous SS activity of cerebellar PCs in urethane-anesthetized mice by *in vivo* electrophysiological recording and pharmacological methods. Cell-attached recordings from PCs showed that micro-application of CRF in cerebellar cortical molecular layer induced a dose-dependent increase in SS firing rate in the absence of GABA_A_ receptor activity. The CRF-induced increase in SS firing rate was completely blocked by a nonselective CRF receptor antagonist, α-helical CRF-(9–14). Nevertheless, application of either a selective CRF-R1 antagonist, BMS-763534 (BMS, 200 nM) or a selective CRF-R2 antagonist, antisauvagine-30 (200 nM) significantly attenuated, but failed to abolished the CRF-induced increase in PCs SS firing rate. *In vivo* whole-cell patch-clamp recordings from PCs showed that molecular layer application of CRF significantly increased the frequency, but not amplitude, of miniature postsynaptic currents (mEPSCs). The CRF-induced increase in the frequency of mEPSCs was abolished by a CRF-R2 antagonist, as well as protein kinase A (PKA) inhibitors. These results suggested that CRF acted on presynaptic CRF-R2 of cerebellar PCs resulting in an increase of glutamate release through PKA signaling pathway, which contributed to modulation of the cerebellar PCs outputs *in Vivo* in mice.

## Introduction

Corticotropin releasing factor (CRF) is a 41-amino acid peptide originally isolated from sheep brain (Vale et al., 1981). CRF is synthesized and secreted in many regions of the central nervous system, and is distributed in the hypothalamus, cerebral cortex, amygdala and spinal cord (Luo et al., 1994). In the mammalian brain, CRF is released following stress and subsequently stimulates the release of adrenocorticotropic hormone from the anterior pituitary, which plays a critical role in the coordination of endocrine and behavioral responses to stress (Vale et al., 1981; Antoni, 1986; Luo et al., 1994).

In mammalian cerebellar cortex, climbing fibers produce and release CRF onto Purkinje cells (PCs; Palkovits et al., 1987). The release of CRF from climbing fibers can be reliably induced by direct electrical or chemical stimulation of the inferior olive, as well as by stimulation of specific sensory afferents (Barmack and Young, 1990; Tian and Bishop, 2003). CRF binds to CRF receptors, consequently modulating spontaneous and glutamate-induced activity in cerebellar PCs (Fox and Gruol, 1993). Two types of CRF receptors, CRF-R1 and CRF-R2, have been identified as G-protein-coupled receptors (Chen et al., 1993). CRF binds to CRF-R1 with high affinity, but has low affinity for CRF-R2 (Dautzenberg and Hauger, 2002). Immunohistochemical studies have demonstrated that both CRF-R1 and CRF-R2 are expressed in the adult rodent cerebellum (Bishop, 1990; Bishop et al., 2000; Lee et al., 2004). CRF-R1 immunostaining is present throughout all lobules of the cerebellar cortex, including the primary dendrites and somas of PCs, molecular layer interneurons (MLIs), Golgi cells, Bergmann glial cells and granule cells (Tian et al., 2008; Tao et al., 2009). In contrast, CRF-R2 immunoreactivity has been detected in the molecular layer during the postnatal development of the mouse cerebellum, suggesting that CRF-R2 be expressed on parallel fibers (Lee et al., 2004). Furthermore, the punctate labeling of CRF-R2 has been confirmed in the molecular layer was localized to parallel fibers and their terminals (Tian et al., 2006). Moreover, both CRF-R1 and CRF-R2 were expressed in climbing fibers from post-natal day 3 to the adult rat cerebellum, and CRF-R1 immunoreactivity was concentrated in apical regions of PC somas and later in primary dendrites exhibiting a diffuse cytoplasmic appearance (Swinny et al., 2003). Physiological studies showed that pharmacological activation of CRF-R2 increased the spontaneous firing rate of PCs in cerebellar slices (Bishop et al., 2006; Tao et al., 2009). CRF modulates not only neuronal excitability and membrane properties, but also synaptic transmission in other brain nuclei and cell types (Kirby et al., 2008; Zhao-Shea et al., 2015). CRF dose-dependently modulates excitatory synaptic transmission through CRF-R1 in the noradrenergic nucleus locus coeruleus in slice preparations (Prouty et al., 2017). Recently, it has been reported that CRF increases PC firing rate by modulating sodium and potassium, and hyperpolarizing activated cationic current currents in cerebellar slices (Libster et al., 2015). Up to now, CRF affects neuronal excitability by modulating neuronal membrane properties and synaptic transmission have been well studied, but the synaptic mechanisms of this remain unclear. Therefore, we here studied the effect of CRF on the spontaneous simple-spike (SS) activity of cerebellar PC in urethane-anesthetized mice by *in vivo* electrophysiological recording techniques and pharmacological methods.

## Materials and Methods

### Anesthesia and Surgical Procedures

The anesthesia and surgical procedures have been described previously (Chu et al., 2011). In brief, the experimental procedures were approved by the Animal Care and Use Committee of Yanbian University and were in accordance with the animal welfare guidelines of the U.S. National Institutes of Health. The permit number is SYXK (Ji) 2011-006. HA/ICR mice were bought from the experiment center of Jilin University and housed under a 12 h light:12 h dark cycle with free access to food and water. Either male (*n* = 28) or female (*n* = 23) adult (6–8-week-old) HA/ICR mice were anesthetized with urethane (1.3 g/kg body weight i.p.). A watertight chamber was created and a 1–1.5 mm craniotomy was drilled to expose the cerebellar surface corresponding to Vermis VI–VII. The brain surface was constantly superfused with oxygenated artificial cerebrospinal fluid (aCSF: 125 mM NaCl, 3 mM KCl, 1 mM MgSO_4_, 2 mM CaCl_2_, 1 mM NaH_2_PO_4_, 25 mM NaHCO_3_, and 10 mM D-glucose) with a peristaltic pump (Gilson Minipulse 3; Villiers, Le Bel, France) at 0.4 ml/min. Rectal temperature was monitored and maintained at 37.0 ± 0.2°C using body temperature equipment.

### Electrophysiological Recording and Drug Application

*In vivo* patch-clamp or cell-attached recordings from PCs were performed with an Axopatch-200B amplifier (Molecular Devices, Foster City, CA, USA). The signal of PC spontaneous activity was acquired through a Digidata 1440 series analog-to-digital interface on a personal computer using Clampex 10.3 software. Patch pipettes were made with a puller (PB-10; Narishige, Tokyo, Japan) from thick-wall borosilicate glass (GD-1.5; Narishige). Recording electrodes (4–6 MΩ) contained a solution of the following composition (in mM): potassium gluconate 120, HEPES 10, EGTA 1, KCl 5, MgCl_2_ 3.5, NaCl 4, biocytin 8, Na_2_ATP 4 and Na_2_GTP 0.2 (pH 7.3 with KOH, osmolarity adjusted to 300 mOsm). The electrophysiological recordings from PCs were performed at depths 250–300 μm under pia mater membrane, and identified by regular spontaneous SS accompanied with irregular complex spike (CS; Chu et al., 2011; Liu et al., 2014; Jin et al., 2015). During whole-cell recoding configuration, the series resistances were in a range of 10–40 MΩ, compensated by 80%. Membrane currents were filtered at 2 kHz, digitized at 20 kHz. For recording spontaneous SS activity, gabazine (20 μM) was routinely included in external recording solutions to block GABA_A_ receptor-mediated inhibitory inputs from MLIs. Recordings of miniature postsynaptic currents (mEPSCs) were performed in the presence of a mixture of gabazine (20 μM) and tetrodotoxin (TTX; 1 μM).

The reagents included human/rat CRF (Peptide Institute Inc., Japan); α-helical CRF-(9–14), BMS-763534, 5-Chloro-1-[(1S)-1-cyclopropyl-2-methoxyethyl]-3-[[6-(difluoromethoxy)-2,5-dimethyl-3-pyridinyl]amino]-2(1H)-pyrazinone; antisauvagine-30, GABAzine (SR95531), hydrobromide (6-imino-3-(4-methoxyphenyl)-1 (6H)-pyridazinebutanoic acid hydrobromide), H89, protein kinase A (PKA) inhibitor; and NBQX, (2, 3-dioxo-6-nitro-1,2,3,4-tetrahydrobenzo[f] quinoxaline-7-sulfonamide) were purchased from Sigma-Aldrich (Shanghai, China). KT5720 and chelerythrine chloride were purchased from Tocris (Bristol, UK). CRF was dissolved in ACSF and applied onto the molecular layer above the recorded PCs at 0.1 μl/s for 100 s by a micro pump (KDS-210, KD Scientific, Holliston, MA, USA). The stock solutions of BMS-763534 and KT6720 were dissolved in dimethyl sulfoxide (DMSO). The other drugs were finally dissolved in ACSF, and applied directly onto the cerebellar surface by a peristaltic pump (Gilson Minipulse 3; Villiers, Le Bel, France) at 0.5 ml/min. After a stable cell-attached or whole-cell recording was configured, the baseline was recorded for 100 s then perfusion of chemicals. For completely inhibiting PKA and PKC, H-89, KT5720 and chelerythrine chloride were perfused for 20 min before the next experiments.

### Statistical Analysis

Electrophysiological data were analyzed with Clampfit 10.4 (Molecular Devices, Foster City, CA, USA). Spontaneous SS activity was calculated from a train of interspike intervals recorded for 50 sof baseline, in the presence of drugs (75 s from beginning of CRF application) and Recovery (10 min), respectively. Some data were normalized with baseline and used for further analyses. After the spontaneous CSs were detected, the pauses of SS were calculated between the last spikelet and the first spontaneous SS firing. The frequency and amplitude of mEPSCs were analyzed using MiniAnalysis software (Version 6.0.3; Synaptosoft, Decatur, GA, USA). The original traces of mEPSCs were filtered digitally at 1 kHz. Only synaptic events showing a clearly defined baseline and a peak were used for amplitude analysis. During analysis, the threshold for detection of mEPSCs was set at 3 pA and the period to search an mEPSC was set at 30 ms. All the parameters were maintained constant for an individual recorded neuron in treatments of ACSF, drugs and recovery. Values are expressed as the mean ± SEM One-way and repeated measures ANOVA followed by Tukey’s *post hoc* test or Two-way ANOVA (SPSS software; Chicago, IL, USA) was used to determine the level of statistical significance between groups of data. *P*-values below 0.05 were considered to indicate a statistically significant difference between experimental groups.

## Results

### Effect of CRF on SS Firing Rate of PCs

**Figure 1:**
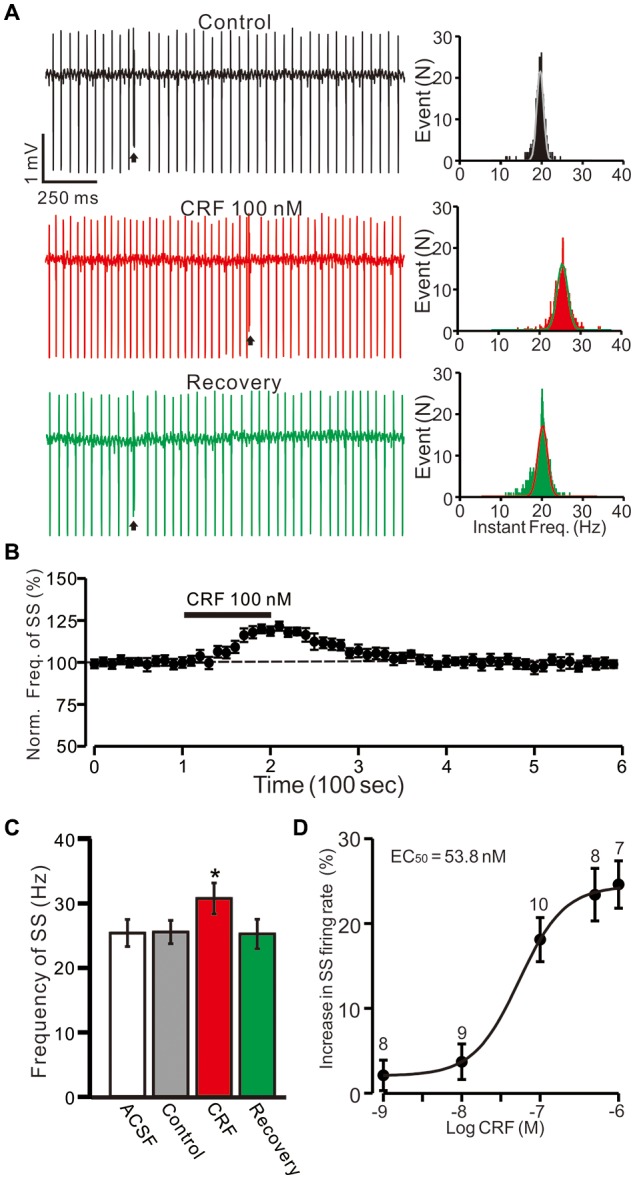
Cerebellar surface application of corticotropin-releasing factor (CRF) increased in spontaneous simple-spike (SS) firing rate of cerebellar Purkinje cells (PCs) in the absence of GABA_A_ receptors activity. **(A)** Left, representative cell-attached recording traces showing the spontaneous SS firing activity of a PC in treatments of control, CRF (100 nM) and washout of CRF (recovery). Right, histograms show the instantaneous frequency of SS firing shown in the Left. Bin = 0.5 s **(B)** Time course of CRF-induced changes in the SS firing rate of the PC. **(C)** Pooled data (*n* = 10 cells) showing the mean frequency of SS firing in aCSF, control (gabazine), CRF (gabazine + CRF) and washout of CRF (recovery). **(D)** The concentration-response curve shows the CRF-induced increases in SS firing rate of cerebellar PCs. The EC_50_ value obtained from the curve was 53.8 nM. The number of the recorded PCs tested for each concentration indicated near the bars. Arrows indicate complex spikes (CSs). *P < 0.05 vs. control.

Under cell-attached recording conditions, a total of 82 cells were identified as cerebellar PCs by exhibiting regular SS and irregular CS activity (Figure 1A, arrows). Molecular layer micro-application of CRF (100 nM) had a small effect on the spontaneous SS firing rate. The mean SS firing rate was 28.7 ± 3.1 Hz, which was not significant compared with control conditions (aCSF: 27.4 ± 3.2 Hz; *P* = 0.52; *n* = 8 cells in 6 mice; data not shown).

### Blockade of GABA_A_ Receptors Activity, CRF Increases PC SS Firing Rate

Since MLIs modulated the spontaneous activity of PCs via GABA_A_ receptors, we examined the effect of CRF on SS activity in the presence of the GABA_A_ receptor antagonist, gabazine (SR95531; 20 μM). Perfusion of gabazine induced a small effect on the spontaneous SS firing rate (Figure 1A). The mean SS firing rate was 25.5 ± 1.8 Hz, which was not significant different than control conditions (aCSF: 25.1 ± 2.1 Hz; *P* = 0.37; *n* = 10 cells in 7 mice; Figure 1C). In the presence of gabazine (20 μM; control), CRF (100 nM) induced increase in the instantaneous frequency of SS firing (Figure 1A). The mean SS firing rate was 30.7 ± 2.4 Hz, which was significantly higher than under control conditions 25.5 ± 1.8 Hz; *P* = 0.031; *n* = 10 cells in 7 mice; Figures 1B,C). The CRF-induced increase in SS firing rate was concentration-dependent (Figure 1D), with a 50% effective concentration (EC_50_) of 53.8 nM. The maximum concentration that increased the spontaneous SS firing rate was 1 μM (24.6 ± 2.8% of baseline; *P* = 0.003 vs. control; *n* = 7 cells in 6 mice). These results indicate that molecular layer application of CRF induces a dose-dependent increase in PC SS firing rate in the absence of GABA_A_ receptor activity.

### Both CRF-R1 and CRF-R2 Are Involved in CRF-Induced Increase of PC SS Firing Rate

**Figure 2:**
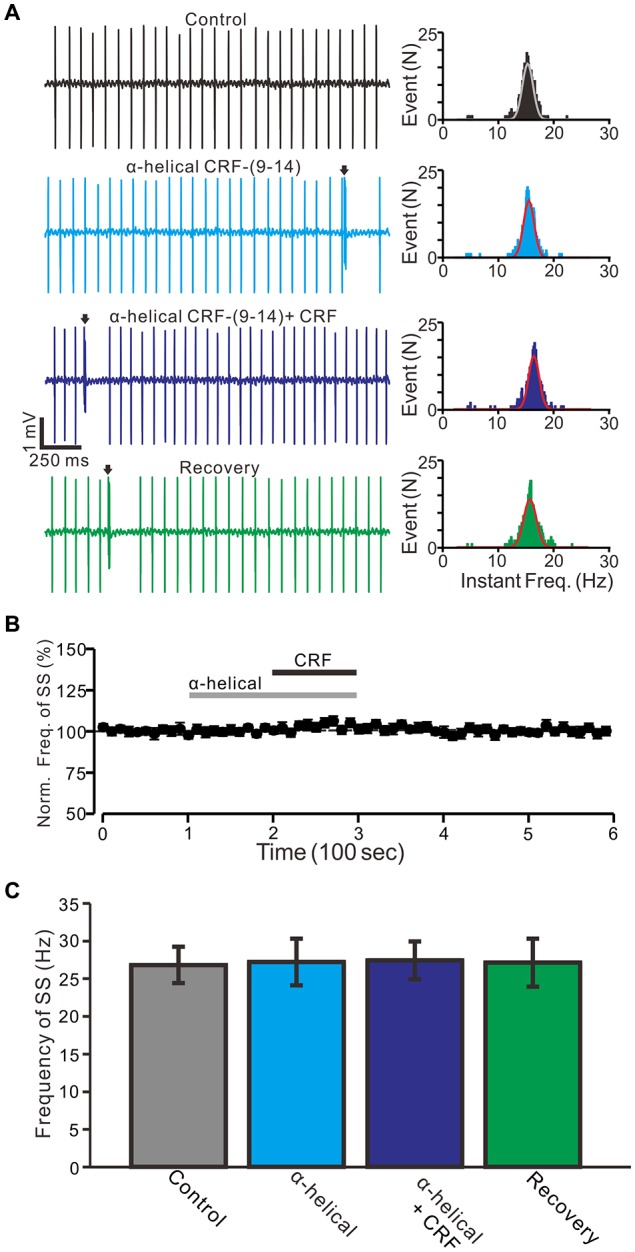
CRF-induced increase in spontaneous SS firing rate of cerebellar PCs was blocked by a non-selective CRF-Rs antagonist, α-helical CRF-(9–14; α-helical). **(A)** Left, representative traces showing the spontaneous SS firing activity of a PC in control, α-helical (1 μM), α-helical + CRF (100 nM) and washout of CRF (recovery). Right panel shows the instantaneous frequency of the SS firing shown in the left. Bin = 0.5 s **(B)** Time course of the PC SS firing rate in control, α-helical, α-helical + CRF and recovery. **(C)** Pooled data showing the normalized frequency of SS firing in control, α-helical, α-helical + CRF and washout of CRF (recovery). Arrows indicate CSs. *n* = 6 cells.

Bath application of an non-selective CRF-Rs antagonist, α-helical CRF-(9–14) (1 μM) for 200 s did not significantly change the SS firing rate of PCs (Figures 2A,B), with a normalized SS firing rate of 98.5 ± 4.8% of control (100.0 ± 2.7%; *P* = 0.72; *n* = 6 cells in 6 mice; Figure 2C). In the presence of α-helical CRF-(9–14), micro-application of CRF failed to increase SS firing rate, with the normalized SS firing rate being 103.3 ± 3.5% of control (98.5 ± 4.8%; *P* = 0.31; *n* = 6 cells in 6 mice; Figures 2B,C). These results indicate that CRF increases the SS firing rate of PCs via activation of CRF receptors.

**Figure 3:**
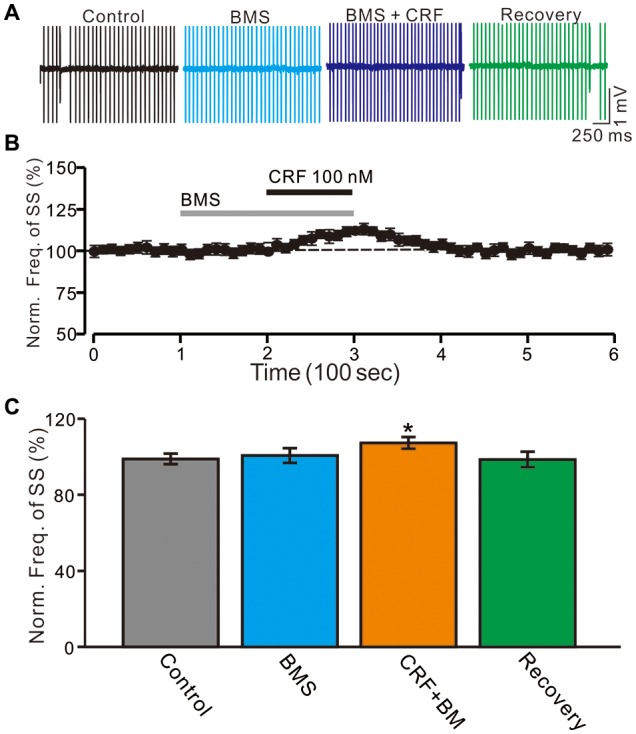
Blockade CRF-R1 failed to abolish the CRF-induced increase in spontaneous SS firing rate of cerebellar PCs. **(A)** Representative cell-attached recording traces showing the spontaneous SS firing activity of a PC in control, BMS-763534 (BMS, 100 nM), BMS + CRF (100 nM) and washout of CRF (recovery). **(B)** Time course of the PC SS firing rate in control, BMS-763534 (BMS, 100 nM), BMS + CRF (100 nM) and washout of CRF (recovery). **(C)** Pooled data showing the normalized frequency of SS firing in in control, BMS-763534 (BMS, 100 nM), BMS + CRF (100 nM) and washout of CRF (recovery). *P < 0.05 vs. control or CRF.

Furthermore, we employed a selective CRF-R1 antagonist, BMS-763534 (BMS, 200 nM) to examine whether the CRF-induced increase in the PC SS firing rate via activation of CRF-R1. Bath administration of BMS for 200 s did not significantly change PC SS firing rate, with a normalized SS firing rate of 101.8 ± 3.9% of control (100.0 ± 2.7%; *P* = 0.68; *n* = 6 cells in 5 mice; Figures 3A,B). In the presence of BMS-763534, micro-application of CRF still induced an increase of SS firing rate (normalized SS firing rate 108.5 ± 3.1% of control, 100.0 ± 2.8%; *P* = 0.04; *n* = 6 cells in 5 mice; Figure 3C). The results indicate that activation of CRF-R1 contributes to CRF-induced increases in PC SS firing rate.

**Figure 4:**
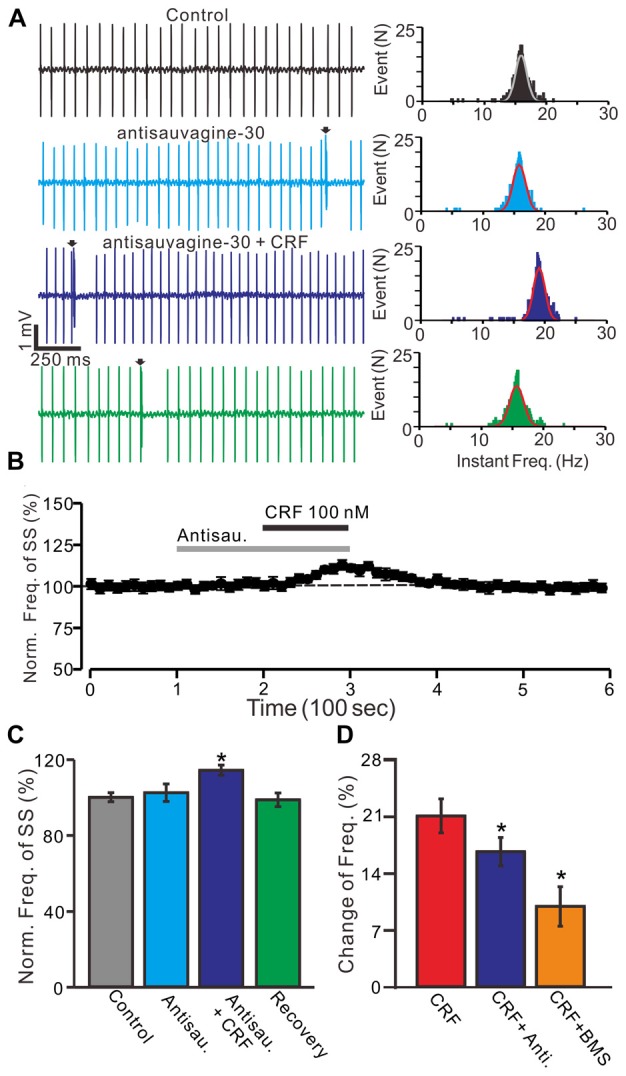
Blockade CRF-R2 failed to prevent the CRF-induced increase in spontaneous SS firing rate of cerebellar PCs. **(A)** Left, representative cell-attached recording traces showing the spontaneous SS firing activity of a PC in control, antisauvagine-30 (200 nM), antisauvagine-30 (200 nM) + CRF (100 nM) and washout of CRF (recovery). Right panel shows the instantaneous frequency of the SS firing shown in the left. Bin = 0.5 s **(B)** Time course of the PC SS firing rate in control, antisauvagine-30, antisauvagine-30 + CRF and recovery. **(C)** Summary of data showing the normalized frequency of SS firing in control, antisauvagine-30, antisauvagine-30 + CRF and washout of CRF (recovery). **(D)** Bar graph showed the change of SS frequency in the treatments of CRF, CRF + antisauvagine, and CRF (100 nM) + BMS. *P < 0.05 vs. control or CRF. Arrows indicate CSs. *n* = 6 cells.

Moreover, we used a selective CRF-R2 antagonist, antisauvagine-30 (200 nM) to determine whether the CRF-induced increase in PC SS firing rate was involved in CRF-R2. Bath administration of antisauvagine-30 for 200 sdid not significantly change the SS firing rate of PCs (normalized SS firing rate 102.4 ± 4.6% of control; 100.0 ± 2.4%; *P* = 0.74; *n* = 6 cells in 6 mice; Figures 4A–C). In the presence of antisauvagine-30, micro-application of CRF still induced an increase in SS firing rate, with a normalized SS firing rate of 114.3 ± 2.7% of control (102.4 ± 4.6%; *P* = 0.037; *n* = 6 cells in 6 mice; Figures 4B,C). Notably, blockade of CRF-R1, CRF increased SS firing rate by 8.5 ± 2.1% of control, which was significantly weaker than controls (18.1 ± 1.8%; *P* = 0.037; *n* = 6 cells in 5 mice; Figure 4D), and blockade of CRF-R2, CRF increased SS firing rate by 14.3 ± 1.5% of control, which was also significantly lower than controls (18.1 ± 1.8%; *P* = 0.037; *n* = 6 cells in 6 mice; Two-way ANOVA Figure 4D).

### Effect of CRF on the Activity Spontaneous Complex Spikes (CSs)

**Figure 5:**
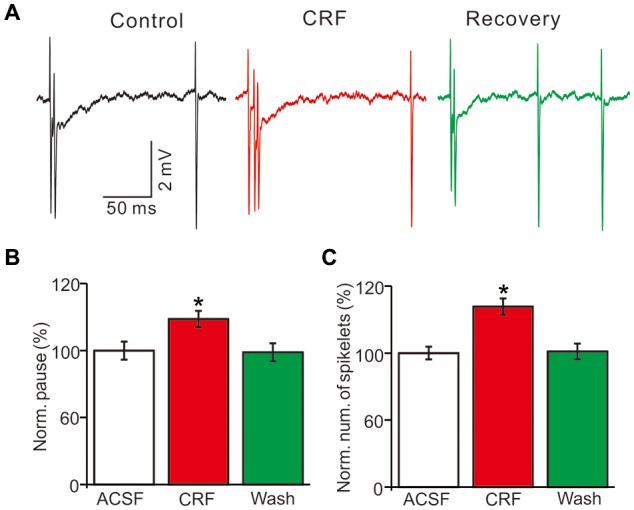
Effects of CRF on the spontaneous CSs activity of cerebellar PCs.** (A)** Representative cell-attached recording traces showing the spontaneous CSs of a PC in treatments of control, CRF (100 nM) and washout of CRF (recovery). **(B)** Summary of data (*n* = 10 cells) showing the normalized pause of SS firing in control, CRF (100 nM) and washout of CRF (recovery). *P < 0.05 vs. control. **(C)** Bar graph shows the normalized number of spikelets in control, CRF (100 nM) and washout of CRF (recovery). *P < 0.05 vs. control. *n* = 10 cells.

**Figure 6:**
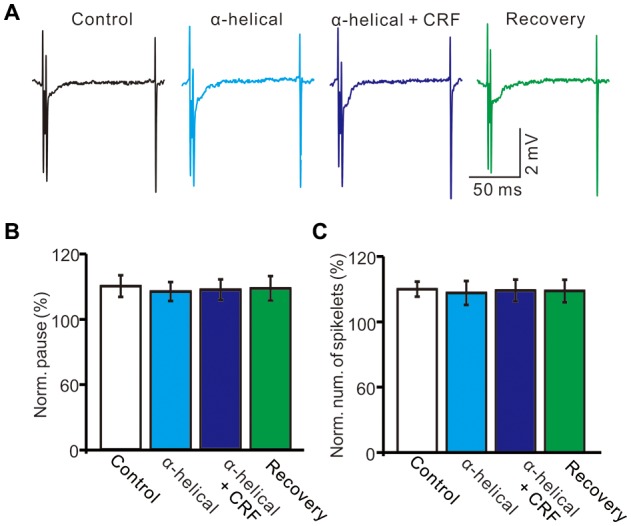
α-helical CRF-(9–14) abolished the effect of CRFon the spontaneous CSs activity of cerebellar PCs. **(A)** Representative cell-attached recording traces showing the spontaneous CSs of a PC in treatments of control, α-helical CRF-(9–14), α-helical CRF-(9–14) + CRF (100 nM) and recovery. **(B)** Summary of data (*n* = 8 cells) showing the normalized pause of SS firing in control, α-helical CRF-(9–14), α-helical CRF-(9–14) + CRF (100 nM) and recovery. **(C)** Bar graph shows the normalized number of spikelets in control, α-helical CRF-(9–14), α-helical CRF-(9–14) + CRF (100 nM) and recovery. *n* = 8 cells.

In the presence of CRF (100 nM), the normalized pause of SS firing was 123.5 ± 6.1% of control (100.0 ± 6.7%; *P* = 0.026; *n* = 10 cells in 7 mice; Figures 5A,B), and the normalized number of spikelets was 134.6 ± 6.3% of control (100.0 ± 4.7%; *P* = 0.015; *n* = 10 cells in 7 mice; Figures 5A,C). However, application of α-helical CRF-(9–14; 1 μM) for 200 s did not significantly change the CSs-evoked pause of SS firing or the number of spikelets (Figure 6). In the presence of α-helical CRF-(9–14), the normalized SS firing pause was 96.7 ± 5.8% of control (100.0 ± 6.6%; *P* = 0.65; *n* = 8 cells in 8 mice; Figures 6A,B), and the normalized number of spikelets was 97.7 ± 7.3% of control (100.0 ± 4.6%; *P* = 0.73; *n* = 8 cells in 8 mice; Figures 6A,C). In the presence of α-helical CRF-(9–14), application CRF failed to increases in CSs-evoked pause of SS (Figures 6A,B) and number of spikelets (Figures 6A,C).

### CRF Increased Presynaptic Excitatory Inputs of Cerebellar PCs

**Figure 7:**
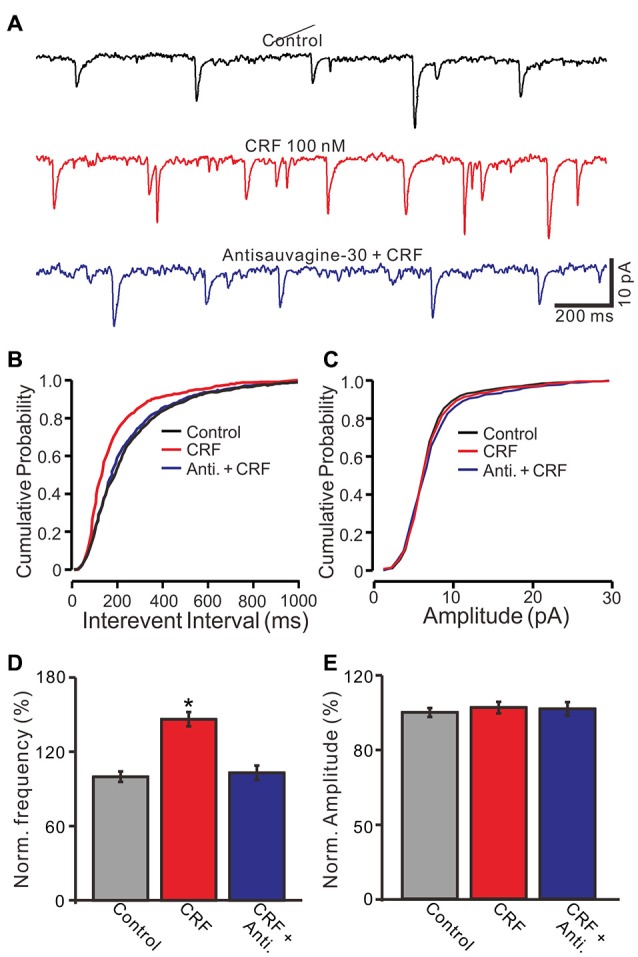
CRF increased the frequency of miniature postsynaptic currents (mEPSCs) in cerebellar PCs via CRF-R2. **(A)** Representative membrane current traces of a cerebellar PC recorded in control (gabazine 20 μM + TTX 1 μM), CRF (100 nM) and antisauvagine-30 (200 nM) + CRF (100 nM). **(B)** Cumulative probability-interevent interval curve of mEPSCs in control, CRF and antisauvagine-30 + CRF. **(C)** Cumulative probability-amplitude curve of mEPSCs in control, CRF and antisauvagine-30 + CRF. **(D)** Summary of the normalized mEPSCs frequency of the PCs in control, CRF and antisauvagine-30 + CRF. (*n* = 6). **(E)** Pooled data showing the normalized mEPSCs amplitude of the PCs in control, CRF and antisauvagine-30 + CRF. *n* = 6. *P < 0.05 vs. control.

*In vivo* patch-clamp recordings showed that cerebellar molecular layer micro-application of CRF significantly decreased the interevent interval of mEPSCs, and shifted the cumulative probability-interevent interval curve of mEPSCs to the left (normalized mean frequency, 146.5 ± 5.7% of control; 100.0 ± 4.2%; *P* = 0.003; *n* = 6 cells in 5 mice; Figures 7A–D). Application of antisauvagine-30 did not change the frequency and amplitude of mEPSCs (not shown). However, the CRF-induced decrease of mEPSC interevent interval was not observed in the presence of antisauvagine-30 (200 nM; Figures 7A,B). In the presence of antisauvagine-30 and CRF, the normalized mean frequency was 103.2 ± 5.9% of control (100.0 ± 4.2%; *P* = 0.65; *n* = 6 cells in 5 mice; Figure 7D). In addition, micro-application of CRF did not significantly change the cumulative probability-amplitude curve (Figures 7A,C) or the mEPSC amplitude, with a normalized mean amplitude of 102.5 ± 3.1% of control (101.8 ± 3.6%; *P* = 0.81; *n* = 6 cells in 5 mice; Figure 7E). These results indicate that CRF increases the frequency of mEPSCs via activation of CRF-R2, suggesting that activation of presynaptic CRF-R2 contributes to the excitation of cerebellar PCs.

**Figure 8:**
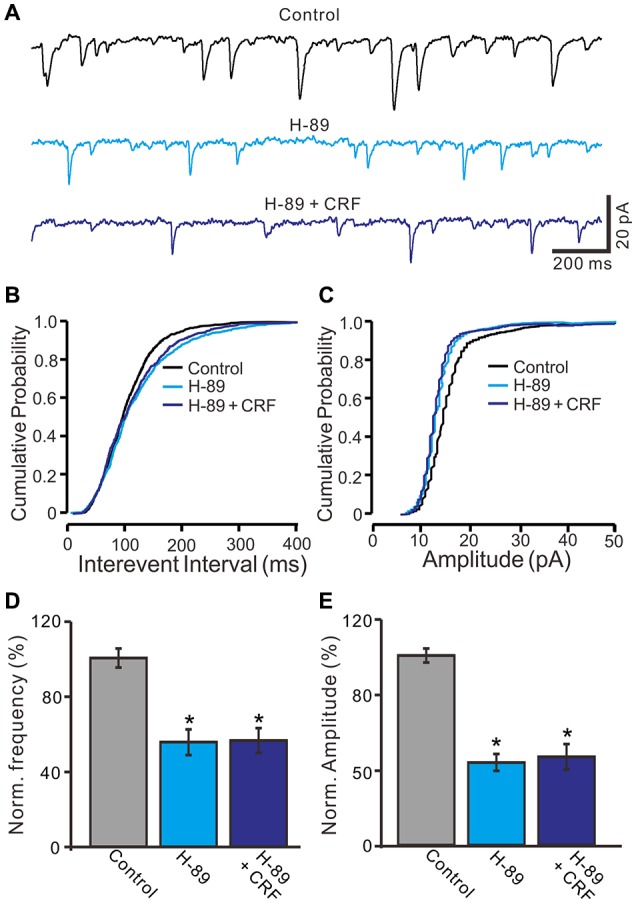
Protein kinase A (PKA) inhibitor, H-89 abolished the CRF-increased frequency of mEPSCs in cerebellar PCs. **(A)** Representative mEPSCs of a cerebellar PC recorded in control (gabazine 20 μM + TTX 1 μM), H-89 (10 μM) and H-89 + CRF (100 nM). **(B)** Cumulative probability-interevent interval curve of mEPSCs in control, H-89 and H-89 + CRF. **(C)** Cumulative probability-amplitude curve of mEPSCs in control, H-89 and H-89 + CRF. **(D)** Summary of the normalized mEPSCs frequency of the PCs in each treatment. **(E)** Pooled data showing the normalized mEPSCs amplitude of the PCs in each treatment. *n* = 7. *P < 0.05 vs. control.

**Figure 9:**
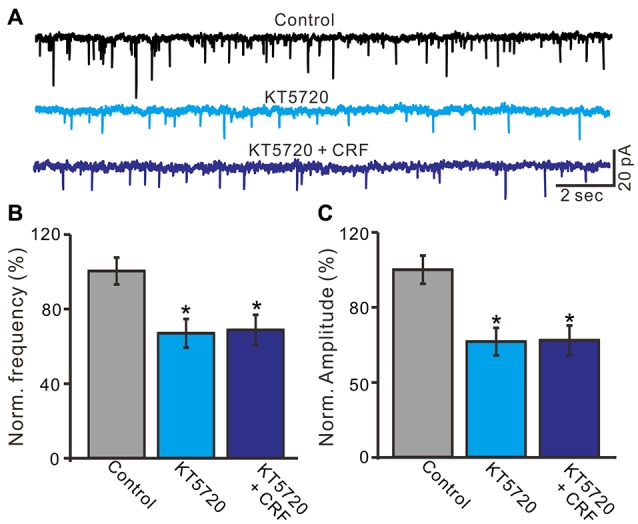
A specific PKA blocker, KT5720 prevented the CRF-increased frequency of mEPSCs in cerebellar PCs. **(A)** Representative mEPSCs of a cerebellar PC recorded in control (gabazine 20 μM + TTX 1 μM), KT5720 (1 μM) and KT5720 + CRF (100 nM). **(B)** Summary of the normalized mEPSCs frequency of the PCs in each treatment. **(C)** Pooled data showed the normalized mEPSCs amplitude of the PCs in each treatment. *n* = 5. *P < 0.05 vs. control.

CRF-Rs couple to Gsα, resulting in the activation of adenylyl cyclase and generation of the second messenger cyclic AMP, and further stimulates PKA or PKC to phosphorylate downstream targets in the cytosol and nucleus (Hauger et al., 2003, 2009; Tao et al., 2009). Therefore we examined whether the CRF-induced increase in mEPSCs via PKA or PKC signaling pathways. Application of PKA inhibitor, H-89 for 10 min, not only increased the interevent interval of mEPSCs (Figures 8A,B), but also abolished the CRF-induced increase in the frequency of mEPSCs. The normalized mean frequency of mEPSCs was 55.4 ± 6.8% (H-89) and 56.3 ± 6.5% (H-89 + CRF) of control (100.0 ± 5.1%; *P* < 0.0001; *n* = 7 cells in 4 mice; Figure 8D). In addition, inhibition of PKA induced a decrease in the amplitude of mEPSCs (Figures 8A,C). The normalized mean amplitude of mEPSCs was 43.6 ± 4.5% (H-89) and 46.5 ± 6.7% (H89 + CRF) of control (100.0 ± 3.7%; *P* < 0.0001; *n* = 7 cells in 4 mice; Figure 8E). Further, we used a more specific PKA inhibiter, KT5760 to determine whether CRF-induced increase in mEPSCs frequency via PKA signaling cascade. Perfusion of KT5720 (1 μM) for 10 min, completely prevented the CRF-induced increase in the frequency of mEPSCs (Figure 9A). The normalized mean frequency of mEPSCs was 66.7 ± 7.6% (KT5720) and 68.5 ± 8.1% (KT5720 + CRF) of control (100.0 ± 7.2%; *P* < 0.001; *n* = 5 cells in 5 mice; Figure 9B). The normalized mean amplitude of mEPSCs was 61.6 ± 7.4% (KT5720) and 62.3 ± 7.9% (KT5720 + CRF) of control (100.0 ± 7.5%; *P* < 0.001; *n* = 5 cells in 5 mice; Figure 9C). In addition, application PKC inhibitor, chelerythrine (50 μM) failed to prevent the CRF-induced increase in frequency of mEPSCs (not shown). These results indicate that the CRF-induced an increase in frequency of mEPSCs depends on activation of PKA cascade rather than activation of PKC signaling pathway.

## Discussion

In this study, we demonstrated that molecular layer application of CRF induces a dose-dependent increase in PC SS firing rate via activation of both CRF-R1 and CRF-R2. Antagonism of CRF-R1 or CRF-R2 significantly attenuated the CRF-induced increase in SS firing rate of the PCs. *In vivo* whole-cell patch-clamp recordings showed that CRF-induced increase in the frequency of mEPSCs was prevented by CRF-R2 antagonist, as well as PKA inhibitors. Our results suggested that CRF acted on presynaptic CRF-R2 of cerebellar PCs resulted in an increase of glutamate release through PKA signaling pathway, which contributed to modulation of PCs outputs *in vivo* in mice.

### CRF Modulates Cerebellar Function and PC SS Firing Activity by Activation of Both CRF-R1 and CRF-R2

In the cerebellar cortex CRF is released from climbing fibers to PCs during direct electrical or chemical stimulation of the inferior olive, as well as by stimulation of sensory afferents (Palkovits et al., 1987; Barmack and Young, 1990; Tian and Bishop, 2003), and modulates spontaneous and glutamate-induced activity in cerebellar PCs (Fox and Gruol, 1993). Consistent with previous studies (Bishop et al., 2000; Dautzenberg and Hauger, 2002; Libster et al., 2015), our results show that molecular layer micro-application of CRF dose-dependently increases the SS firing rate of cerebellar PCs in the absence of GABA_A_ receptor activity.

Both CRF-R1 and CRF-R2 are expressed in the adult rodent cerebellum (Bishop et al., 2000; King and Bishop, 2002; Lee et al., 2004; Tian et al., 2006). CRF-R1 is distributed over the somas and primary dendrites of the PCs in the molecular layer of the cerebellar cortex (Bishop et al., 2000; King and Bishop, 2002), whereas CRF-R2 has been found throughout the molecular layer in cerebellar cortex, including parallel fibers and their terminals (Bishop et al., 2000; Jedema and Grace, 2004). In this study, the CRF-induced increase in SS firing rate of PCs was abolished by a non-selective CRF-Rs antagonist, indicating that CRF modulates the output of PCs via CRF-Rs. A previous study demonstrated that CRF-R2α mRNA, but not that of CRF-R1 or CRF-R2β, is endogenously expressed in the rat cerebellum (Tao et al., 2009). In this study, a selective CRF-R1 antagonist significantly attenuated the CRF-induced increase in SS firing rate of PCs, suggesting that CRF-R1 is expressed on PCs and modulates SS activity. In addition, CRF-R2 has been found in a subpopulation of PCs and Bergmann glial cells in the cerebellum (Swinny et al., 2003; Lee et al., 2004; Bishop et al., 2006). Under *in vitro* conditions, it has been demonstrated that a selective CRF-R2α agonist increases the SS firing rate of PCs, and this response can be blocked by a CRF-R2α-specific antagonist (Bishop et al., 2006). This indicates that CRF-R2α is present in the cerebellum and modulates the SS firing rate of PCs. Activation of CRF-R2 has been also found to inhibit P-type Ca^2+^ currents and to increase the spontaneous firing frequency of PCs in cerebellar slices (Tao et al., 2009). Our results show that CRF-induced increases in SS firing rate are significantly inhibited by a selective CRF-R2 antagonist, indicating that activation of CRF-R2 contributes to the CRF-induced increase in PC SS firing rate.

### Ionic and Synaptic Mechanisms of CRF-Induced Increase in PC SS Firing Rate

CRF is present in specific populations of climbing fibers and mossy fibers in particular, the lateral aspect of vermal lobules VII and VIII in cerebellar cortex (Bishop, 1990; Bishop et al., 2000). Extracellular application of CRF enhances the spontaneous and excitatory effects of both aspartate and glutamate, suggesting that CRF acts as a neuromodulator in cerebellar circuitry (Bishop, 1990; Bishop et al., 2000). Under *in vitro* conditions, CRF dose-dependently reduces the amplitude of the afterhyperpolarization, but does not significantly alter membrane properties of the PCs, suggesting that CRF regulates the activity of PCs via an indirect pathway (Fox and Gruol, 1993). In addition, CRF coupled with CRF-R1 results in depolarization of noradrenergic nucleus locus coeruleus neurons through a cyclic AMP-dependent reduction in potassium conductance (Jedema and Grace, 2004; Reyes et al., 2007). Recently, it has been demonstrated that CRF dose-dependently modulates excitatory synaptic transmission in the noradrenergic nucleus locus coeruleus, suggesting that CRF affects neuronal activity via modulation of synaptic transmission (Prouty et al., 2017). We here showed that cerebellar molecular layer micro-application of CRF significantly increased the mEPSCs frequency, but not the amplitude. Consistent with previous studies (Lee et al., 1993; Lawrence et al., 2002; Lewis et al., 2002), the results indicated that CRF-enhanced presynaptic glutamate release results in an increase in mEPSC frequency, as well as contributing to CRF-induced increases in SS firing activity. Notably, the CRF-induced increase in mEPSC frequency was completely blocked by a selective CRF-R2 antagonist, indicating that CRF increases the frequency of mEPSCs via the activation of CRF-R2 located at presynaptic sites. Cerebellar PC receives numerous parallel fiber excitatory inputs, and CRF-R2 has been found in parallel fibers and their terminals (Bishop et al., 2000; Jedema and Grace, 2004). Thus, activation of parallel fiber presynaptic CRF-R2 could contribute to an increase in mEPSCs in cerebellar PCs. However, we cannot exclude the possibility that the mEPSC increase is mediated by glutamate release from climbing fiber excitatory inputs, since CRF-R2 might be expressed on climbing fiber terminals. It has been reported that CRF facilitates norepinephrine release through a presynaptic facilitation mechanism in the dentate gyrus (Lee et al., 1993). CRF-R2 has been found on afferent terminals of the vagus nerve in the nucleus of the tractus solitarius (Lawrence et al., 2002), and on parallel fiber terminals in the cerebellar cortex (Tian et al., 2006). CRF has been found to have an indirect excitatory effect on dorsal vagal neurons via the activation of CRF-R2 at presynaptic sites (Lewis et al., 2002). Although it has been found that CRF-R2 is localized presynaptically in the cerebellar cortex (Tian et al., 2006) and activation of CRF-R2 increases PC firing rate in cerebellar cortical slices (Bishop et al., 2006; Tao et al., 2009), there has been no direct evidence to show the effect of CRF on cerebellar presynaptic CRF-R2. Our present results suggest that activation of presynaptic CRF-R2 contributes, at least in part, to an increase in the SS firing rate of cerebellar PCs.

Additionally, CRF has also been shown to modulate neuronal excitability and membrane properties various cell types (Qiu et al., 2005; Kirby et al., 2008; Chu et al., 2012). CRF has direct effects on the dorsal raphe nucleus neurons, eliciting an inward current in 5-hydroxytryptamine neurons via activation of CRF-R2 and in non-5-hydroxytryptamine neurons through CRF-R1 (Kirby et al., 2008). We previously found that CRF depolarizes hypothalamic paraventricular nuclei neurons by activation of hyperpolarization activated inward currents via postsynaptic CRF-R1 (Qiu et al., 2005). In the cerebellar cortex, CRF increases the SS firing rate of PCs, regardless of whether they are firing tonically or switching between firing and quiescent periods (Libster et al., 2015). However, this is associated with a voltage shift of the activation curve of the persistent sodium current and hyperpolarizing-activated current, as well as activation of voltage-dependent potassium current (Libster et al., 2015). Our present results showed that application CRF at 100 nM induced enhance of spontaneous activity, which expressing increases in the number of spikelets and the SS pause. However, the previously study showed that exogenous application of CRF at 1 μM induced reductions in the CF-evoked excitatory postsynaptic current and CS afterhyperpolarization (Schmolesky et al., 2007). The contradict results were considered as the following reasons. The concentrations of CRF was high (1 μM) in the previous study under *in vitro* conditions, but we used low concentration (200 nM) of CRF in the present study in living mice. Moreover, there are many effective factors under *in vivo* conditions, and the concentration of CRF reached to PCs was lower than 200 nM. Therefore, the lower concentration CRF might enhance the CS activity, but high concentration might depress the CF-PC synaptic transmission. In addition, the effects of CRF on cerebellar PC activity might be affected by urethane anesthesia. However, urethane depresses neuronal excitability through activation of barium-sensitive potassium leak conductance, without affecting glutamate-mediated excitatory synaptic transmission or GABAergic inhibitory synaptic transmission (Sceniak and Maciver, 2006).

CRF-Rs couple to Gsα, resulting in the activation of adenylyl cyclase and generation of the second messenger cyclic AMP has been previously demonstrated (Hauger et al., 2003, 2009). Increasing cyclic AMP level further stimulates PKA to phosphorylate downstream targets in the cytosol and nucleus (Hauger et al., 2003, 2009). CRF-R1 signaling to cyclic AMP-PKA pathway contributes to the regulation of synaptic plasticity in hippocampus (Sheng et al., 2008). CRF-R1 upregulates brain-derived neurotrophic factor mRNA levels via the cyclic AMP PKA signaling pathway in cerebellar granular cells (Bayatti et al., 2005). However, CRF-R2 coupling to Gs modulates limbic dopaminergic neurotransmission by stimulating intracellular calcium release via the cyclic APM-PKA signaling pathway (Riegel and Williams, 2008). In this study, we found that inhibition of PKA activity significantly reduced the both minis amplitude and frequency, suggesting that the adenylyl cyclase-cyclic AMP signal-transduction pathway played an important role during the presynaptic neurotransmitter release. Because the PKA phosphorylate proteins synapsin I and II, resulting increases the number of synaptic vesicles in the releasable pool (Greengard et al., 1993). Therefore, inhibition of PKA activity induced a decrease in the amount of phosphorylated synapsins, and caused a decrease in presynaptic glutamate release. Notably, PKA inhibitor not only increased the basal interevent interval of mEPSCs, but also abolished the CRF-induced increase in the frequency of mEPSCs, indicated that CRF increased the frequency of mEPSCs was dependent on activation of PKA pathway. Our results are consistent with previous studies (Bayatti et al., 2005; Riegel and Williams, 2008; Gutknecht et al., 2009), suggesting that CRF-R2 coupling to Gs induces activation of adenylyl cyclase-cyclic AMP signal-transduction, which might lead to phosphorylation of proteins on synaptic vesicles in presynaptic nerve terminals, resulting in an increase in glutamate release onto cerebellar PCs. On the other hand, activation of PKA can phosphorylate several other proteins that are necessary for the vesicle mobilization/priming and exocytosis of synaptic vesicles, and inhibition of basal PKA phosphorylation decreases the synaptic vesicle priming and pool size (Nagy et al., 2004; Maximov et al., 2007). Therefore, we could not occlude the CRF-induced increase in mEPSCs through a difference signaling pathway.

## Author Contributions

Y-ZL, C-PC and D-LQ conceived and designed the experiments. H-WW, J-TZ and B-XL performed the experiments. C-PC, D-LQ and H-WW analyzed the data. S-SS and Y-HB contributed reagents, materials, and analysis tools. C-PC, W-MW and D-LQ wrote the manuscript.

## Conflict of Interest Statement

The authors declare that the research was conducted in the absence of any commercial or financial relationships that could be construed as a potential conflict of interest.
